# A Web-Based Program to Increase Knowledge and Reduce Cigarette and Nargila Smoking Among Arab University Students in Israel: Mixed-Methods Study to Test Acceptability

**DOI:** 10.2196/jmir.2988

**Published:** 2015-02-20

**Authors:** Jumanah Essa-Hadad, Shai Linn, Sheizaf Rafaeli

**Affiliations:** ^1^Bar Ilan UniversityFaculty of Medicine in the GalileeSafedIsrael; ^2^Faculty of Social Welfare and Health StudiesSchool of Public HealthUniversity of HaifaHaifaIsrael; ^3^Faculty of Social Welfare and Health StudiesUniversity of HaifaHaifaIsrael; ^4^Center for Internet ResearchUniversity of HaifaHaifaIsrael

**Keywords:** Web-based intervention, smoking cessation, nargila smoking, Arabs in Israel, university students, tailored feedback

## Abstract

**Background:**

Among Arab citizens in Israel, cigarette and nargila (hookah, waterpipe) smoking is a serious public health problem, particularly among the young adult population. With the dramatic increase of Internet and computer use among Arab college and university students, a Web-based program may provide an easy, accessible tool to reduce smoking rates without heavy resource demands required by traditional methods.

**Objective:**

The purpose of this research was to examine the acceptability and feasibility of a pilot Web-based program that provides tailored feedback to increase smoking knowledge and reduce cigarette and nargila smoking behaviors among Arab college/university students in Israel.

**Methods:**

A pilot Web-based program was developed, consisting of a self-administered questionnaire and feedback system on cigarette and nargila smoking. Arab university students were recruited to participate in a mixed-methods study, using both quantitative (pre-/posttest study design) and qualitative tools. A posttest was implemented at 1 month following participation in the intervention to assess any changes in smoking knowledge and behaviors. Focus group sessions were implemented to assess acceptability and preferences related to the Web-based program.

**Results:**

A total of 225 participants—response rate of 63.2% (225/356)—completed the intervention at baseline and at 1-month poststudy, and were used for the comparative analysis. Statistically significant reductions in nargila smoking among participants (*P*=.001) were found. The intervention did not result in reductions in cigarette smoking. However, the tailored Web intervention resulted in statistically significant increases in the intention to quit smoking (*P*=.021). No statistically significant increases in knowledge were seen at 1-month poststudy. Participants expressed high satisfaction with the intervention and 93.8% (211/225) of those who completed the intervention at both time intervals reported that they would recommend the program to their friends, indicating excellent acceptability and feasibility of the intervention. This was further emphasized in the focus group sessions.

**Conclusions:**

A tailored Web-based program may be a promising tool to reduce nargila smoking among Arab university students in Israel. The tailored Web intervention was not successful at significantly reducing cigarette smoking or increasing knowledge. However, the intervention did increase participants’ intention to quit smoking. Participants considered the Web-based tool to be an interesting, feasible, and highly acceptable strategy.

**Trial Registration:**

Trial Registration: ISRCTN registry ISRCTN59207794; http://www.isrctn.com/ISRCTN59207794 (Archived by WebCite at http://www.webcitation.org/6VkYOBNOJ).

##  Introduction

It is widely known that smoking is the most preventable cause of morbidity and mortality worldwide [1]. Cigarette smoking is a serious public health problem in Israel, particularly among the male Arab population. Data from the Israeli Ministry of Health (2012) indicates that over 52% of Arab males smoke cigarettes [2]. In addition to cigarette smoking, nargila smoking is a phenomenon that has increased significantly over the years among the Arab population in Israel. A survey conducted by the Rikaz Database (2010) using a representative sample revealed that 60.5% of Arabs in Israel have tried using nargila at least once, and about 20% use nargila regularly, daily, or once a week [3]. When examining nargila smoking with respect to age, it is evident that the largest percentage (60.5%) of smokers are 18 to 34 year olds, the age of most college/university students. Females make up a sizable percentage (about 19.6%) of nargila smokers, in comparison to their low representation among cigarette smokers [3]. Multiple studies have shown nargila smoking to be equally dangerous to one’s health as cigarette smoking, linking it with lung disease, cancer of the lung, mouth, and cheek, cardiovascular disease, hypertension, and chronic respiratory disease [4-6].

Well-designed smoking prevention and cessation programs can substantially contribute to global public health. Interventions that can reduce tobacco use, offer global reach, and do so in a cost-effective manner have a tremendous opportunity to reduce the future burden of disease. Several studies have evaluated the effectiveness, feasibility, and acceptability of using computer- and Web-based health interventions to reduce smoking behavior [7-12]. A recent Cochrane review of 28 randomized and quasi-randomized trials on Internet-based interventions for smoking cessation concluded that Internet-based interventions were promising and effective at assisting smoking cessation [13]. The most promising interventions were ones that were tailored to the individuals. Computer-tailored health interventions can be defined as the adaptation of health education materials to one specific person through a largely computerized process [12]. Computer-tailored health programs provide respondents with personalized feedback about their present health behavior and/or behavioral determinants, based on responses reported in a questionnaire. Computer-tailored health interventions may offer a mix of highly professional expertise, typical of individual professional attention (tailored messages), while maintaining the cost-effectiveness of mass communication [14]. Compared with nontailored messages, tailored health messages are more likely to be read and remembered, saved and discussed with others, perceived as interesting and personally relevant, and designed especially for the recipient [15,16].

To date, there are no studies in the literature that have developed, or examined the acceptability and feasibility of, computer-based programs providing tailored health educational feedback among Arabs in Israel. Particularly among the Arab minority in Israel, cigarette and nargila smoking are a serious public health problem. With the dramatic increase of computer and Internet access and use among Arab university students, specifically, and educated Arabs in general, a Web-based program could provide an easy, accessible tool to collect data, increase smoking knowledge, and reduce smoking behavior without the heavy demands of time, manpower, and resources required by traditional methods of data collection and health education.

The aim of this study was to pilot-test the feasibility and acceptability of a Web-based health education program that provides tailored feedback on improving knowledge about cigarette and nargila smoking and changing smoking behaviors of Arab college/university students in Israel. This study aimed to determine if a tailored Web-based program was perceived as an acceptable, preferable, and useful tool among Arab university students.

## Methods

### Study Design and Procedure

We conducted a mixed-methods study utilizing quantitative and qualitative tools to evaluate the feasibility and acceptability of a Web-based smoking cessation program to improve cigarette and nargila smoking knowledge and behaviors among Arab university students in Israel. Quantitative analysis involved a pre-/poststudy design with follow-up measurements after 1 month. Students were recruited to the study using flyers and announcements placed in academic departments, through student message boards, during classes, and through Facebook. Participants were sent an introductory email in Arabic with basic information regarding the questionnaire, the link to the program, and their assigned username and password to allow access to the program. Participants could access the program online from any computer with Internet access.

Participants' responses were automatically saved into the computer database system. This allowed participants to log on multiple times at different sittings, if needed, to complete the questionnaire and receive educational feedback. Following completion of the questionnaire by the participants, responses were saved into the system and could be downloaded into a Microsoft Excel file by the researcher for analysis. Website analytics were used to assess participants’ engagement in the program and length of time that participants used the website.

Email messages were used to remind individuals about follow-up dates for completing the questionnaires after 1 month. Reminders were sent out by email and/or via Facebook messages 2 weeks, 1 week, and 1 day before students were expected to participate in the follow-up session. Participants were given 1 week to complete the online questionnaire. If after 1 week they had not logged on to complete the program, another email reminder was sent to them every other day for a period of 2 weeks. Those participants who did not respond were removed from the follow-up analysis.

Following the online intervention, focus group methodology was utilized to further examine participants’ personal perceptions and opinions regarding the acceptability, appeal, and effectiveness of such a Web-based health program. A focus group guide was developed in a semistructured way to ensure that key questions were addressed and to permit comparisons across groups, while at the same time providing the facilitator with the freedom to follow up on unanticipated topics. During the focus group sessions, participants were also asked to report on how thoroughly they read the educational materials and how interesting and helpful they found the material to be. Five focus group sessions were conducted based on procedures suggested by Krueger [17]. Due to sensitive issues that were expected to arise in the discussion, males and females were separated in the focus group sessions to allow them to discuss issues more comfortably. Participants who completed both the pre- and poststudy questionnaires were randomly recruited to participate in the sessions. To select participants, the researcher used an online random number generator and contacted those chosen via email or phone requesting their participation until the needed number of participants were recruited. All five focus group sessions lasted approximately one and a half hours each. The sessions were moderated by an Arab professional group facilitator, familiar with the health field.

This trial was registered with the ISRCTN registry (ISRCTN59207794).

### Intervention

A Web-based program was developed using the already existing Questions Sharing and Interactive Assignments (QSIA) system, an online assessment system that enables users, teachers, and students to generate, share, and manage knowledge items for learning, teaching, and assessment [18]. The validity and reliability of QSIA has been evaluated by previous research studies [19].

The program, which was in the Arabic language, consisted of two parts: (1) a self-administered online questionnaire, and (2) dissemination of tailored health education material via text and videos. The self-administered online questionnaire consisted of a total of 13 questions on cigarette and nargila smoking behavior and knowledge. The program consisted of a second module with seven questions on demographic information about the participant (ie, age, gender, year of study, subject of study, religion, religiosity, and hometown). The smoking module was only one of four health behaviors of the complete program, the rest of which is not discussed in this paper. All participants gave informed consent before beginning the online program. They were asked to participate in the intervention at baseline, with follow-up after 1 month. The questionnaires that they completed at the follow-up session were identical to the questionnaires at baseline. The module on demographic information was completed only once, at baseline.

After completion of the module, the program was designed to immediately analyze responses and to automatically display on the screen the health educational material in Arabic for the participant to read and watch. The educational material consisted of the following components: (1) introduction, including specific feedback on the respondent's cigarette and nargila smoking behavior and his or her intention to quit smoking or to maintain nonsmoking, (2) educational feedback on the adverse health impact of smoking and a list of potential health risks of smokers (or protective factors for nonsmokers), (3) recommendations with specific actions to help participants quit smoking (based on their intention to quit), and (4) suggestions and tips to cope with difficult social situations, including peer pressure. Various educational YouTube videos showing the adverse health impacts of cigarette and nargila smoking were integrated into the text feedback. The feedback that was given to the tailored-feedback group was done so according to the individual's perceived intention to change certain behaviors, according to the Transtheoretical Stages of Change Model [20]. The feedback was also tailored according to the demographic variables indicated, particularly gender, marital status, and family status. Females received information regarding how smoking can have hazardous implications for future pregnancies, and participants with families received feedback regarding adverse impacts on the health of their children. For example, if an individual in the tailored-feedback intervention group reported that they did not smoke cigarettes or nargila, the feedback given was the following:

Congratulations! You are not a smoker. You are protecting your health and have less chance of developing certain diseases like cancer and heart disease in the future! Keep up the good work!

On the other hand, if the participant reported that they smoked only nargila, for example, they would receive the following recommendation:

It is good that you do not smoke cigarettes. However, by smoking nargila you are putting your health at risk. As a nargila smoker, you are greatly increasing your risk of getting certain diseases like cancer and heart disease in the future. Nargila smoking is just as dangerous as cigarette smoking. It is very important that you quit smoking now to protect your health. It seems that you are ready to quit smoking! You can quit smoking by...(continue with recommendations).

Feedback for cigarette smoking and nargila smoking was given independently. Thus, a participant who did not smoke cigarettes, but smoked nargila would receive positive feedback that they were not a cigarette smoker, and educational feedback regarding nargila smoking.

This feedback was also sent via email message to the address provided by the participant and was given at the baseline session, as well as after the 1-month follow-up session. As part of the feedback and recommendations, there were also links to the educational YouTube videos that the participants could watch.

The intervention was developed with an embedded tracking system—all user activities were logged in a tracking area by username. These database files were accessible through the program website, allowing participants to save answers and complete the program in more than one sitting. The log files contained dates of each individual’s participation in the intervention sessions, and time spent logged into the program.

### Pilot-Testing of the Program

Before the program was administered to the participants, it was pilot-tested for appropriateness to (1) familiarize data collection personnel with the computer program (QSIA), (2) examine online interactions between the participants and the researcher, (3) identify potential problems in the computer process, and (4) modify the questions appropriately to assure cultural appropriateness, user friendliness, and clarity. The program was sent to 25 individuals, including teachers of the Arabic language. These people were then asked to provide feedback regarding clarity of the questions and health educational materials, grammar and spelling, and other administrative issues, such as the correct ordering of questions, and whether appropriate health education was received in the tailored-feedback intervention. After receiving the feedback from the pilot participants, either through email, phone conversation, or face-to-face meeting, changes were made to the program before administering it to the participants. The data from the pilot study was not used in the analysis.

### Target Population and Sampling Procedure

Male and female Arab students attending colleges and universities in Israel from 2007 to 2010 were recruited to participate in this study. The study sample chosen was not meant to be representative of the entire Arab population in Israel—the study was not aimed at describing the whole Arab population, but rather to study an influential group that can affect the future. This research will assess whether this type of program is applicable to this particular group. Data are lacking on the health knowledge, attitudes, and practices of young, educated Arab adults. In general, this group is often overlooked because its members are regarded as a healthy, invulnerable population. However, university students are likely to engage in risky health behaviors, including smoking, which can significantly impact their future health. University students are a very influential group who are likely to become the future leaders of their communities. Knowledge obtained from studying this group may have an impact on the whole Arab population. Increasing health knowledge and awareness by this target population is likely to have a significant impact on others. For example, many of these students will become the future educators and leaders of the next generation. It is likely that these students either have young children or will soon become parents and raise families. Increasing their awareness and knowledge regarding health issues and health-related behaviors empowers them to promote healthy behaviors among their future students, spouses, children, and families.

Potential participants received email messages in Arabic prior to the study, explaining its purpose and general procedure. All individuals who agreed to participate provided online consent prior to completing the online questionnaire. Once informed consent was given, participants were sent an email message containing their assigned username and password, and a link to enter the online program.

### Participant Recruitment

Students were recruited to the study using flyers and announcements placed in academic departments and student Internet message boards in various colleges and universities throughout Israel. Social media was also utilized as a means of recruiting participants. An event page on Facebook was created—individuals that “liked” the page were asked by the researcher to spread the word regarding the need for students by sending messages to their friends. Finally, the researcher requested permission from lecturers to enter during certain classes to recruit participants. Interested participants were asked to provide their contact details, including mobile phone number and email address.

The eligibility criteria included the following: (1) an Arab studying at a college or university in Israel, (2) 18 years of age or older, (3) has access to the Internet either at home or at their corresponding college or university, and (4) agrees to provide informed consent for participation.

### Measures

#### Outcome Measures

Our primary outcome measure was self-reporting of cigarette and nargila smoking behavior. Smoking behavior was assessed by asking the questions*,* “In the past 7 days, have you smoked a cigarette?” and ;“ In the past month, have you smoked nargila at least once a week?” The answer options were *Yes* or *No, not even a puff*.

Increases in cigarette and nargila smoking knowledge was also an outcome measure. To measure participants’ knowledge regarding cigarette and nargila smoking at baseline, we administered two questions on the health consequences of smoking. Increase in knowledge was assessed through completion of the self-administered questionnaire.

Acceptability and preference of the program were additional outcome measures that were assessed both from the online questionnaire and through the focus group sessions. Participants were asked to assess their satisfaction with the Web-based intervention as *very satisfied*, *satisfied*, *neutral*, *dissatisfied*, and *very dissatisfied*. In addition, they were asked if they would recommend the intervention to a friend.

Secondary outcome measures included intention to quit smoking, reason for wanting to quit, and seeking of professional help to quit smoking. Intention to quit smoking was assessed by *Yes* or *No* answers to the question, “Do you intend to quit smoking cigarettes/nargila in the next 6 months?” Participants were also asked about the reasons they had for wanting to quit smoking, as well as how they have tried to quit in the past (eg, seeking professional help, support from family/friends).

#### Baseline Measures

Sociodemographic characteristics, including age, gender, year of study, subject of study, religion, religiosity, and hometown, were collected from all the participants during the baseline questionnaire.

### Statistical Analysis

Descriptive statistics were first calculated to identify the characteristics of the participants and frequencies of behavior and knowledge change. Pearson's chi-square test was used to determine statistical differences between the pre- and poststudy questionnaires for categorical variables. Only participants who completed both pre- and poststudy questionnaires were used in the comparative analysis. Measures of acceptability and satisfaction were summarized using counts and sample proportions. Given the preliminary nature of the study and small sample size of smokers, multivariate analyses were not conducted.

In order to analyze the focus group sessions, all sessions were audiotaped and then transcribed. Notes were kept during the sessions to capture the nonverbal “mood of the moment” that could not be documented through the recordings. Following each focus group session, members of the research team conducted a debriefing to identify issues that could affect analysis, such as domineering or quiet members [21]. The completed transcripts were compared with handwritten notes, and any inaudible phrases or gaps in the tapes were noted. Verification of the accuracy of the transcripts was achieved by randomly cross-checking the transcripts against the tapes.

The results from the focus groups were analyzed using thematic analysis of the transcripts. This was done by organizing the statements from the focus group sessions into categories on the basis of themes (or concepts) for each of the focus group questions that were asked. Concepts were then linked together as opposites or as sets of similar categories, which were then made into theoretical statements. A selective coding template was developed based on major data themes—each theme was given a different coding letter.

## Results

### Participation and Demographics

At baseline, 356 participants completed the intervention. The completion rate was 63.2% (225/356) at the 1-month follow-up session. Only participants who completed both the pre- and poststudy questionnaires were used in the analysis.

The mean age of respondents was 25 years (SD 5). More than two-thirds of all respondents were female (155/225, 68.9%). There were slightly more Muslim students that Christians—47.1% (106/225) versus 43.1% (97/225), respectively. The majority (164/225, 72.9%) of students reported that they were religious. Most students were undergraduate-level students (165/225, 73.3%). About 70.2% (158/225) of students were single. These sociodemographic factors may have an important impact on how the participants use and perceive computers, which could directly impact the results of the intervention. Thus, it is important that these factors were controlled for and considered throughout the results and discussion. Demographic characteristics of the participants are presented in Table 1.

### Cigarette and Nargila Smoking Behavior and Knowledge

At baseline, 22.2% (50/225) of participants reported that they smoked at least one cigarette in the past week. Participation in the tailored-feedback intervention did not have a significant impact on cigarette smoking behavior—the percentage of smokers remained approximately the same at the 1-month poststudy session (see Table 2).

More than 58.2% (131/225) of participants reported that they smoked nargila at baseline. At the 1-month follow-up session after the intervention, this decreased significantly to 22.2% (50/225) of participants reporting they smoked nargila on a regular basis (χ^2^_1_=60.6, *P*=.001).

More than half of participants who smoked cigarettes (29/50, 58%), indicated that they had recently tried to quit smoking. The percentage of participants in the tailored-feedback intervention who tried to quit smoking increased to 73% (33/45) at the 1-month poststudy session. At 1-month poststudy, there was a statistically significant increase in intention to quit smoking from 58% (29/50) to 80% (36/45) (χ^2^_1_=5.3, *P=*.021). At this time, the number of smokers who sought professional help to quit smoking increased from 68% (34/50) to 76% (34/45), however, this increase was not statistically significant.

The primary reason given for trying to quit smoking was to improve health status. There was no significant change over time related to this. The majority of participants (156/225, 69.3%) were knowledgeable that smoking, even for a short period of time (1 to 2 years), had an adverse impact on health. There was a slight increase in this knowledge to 73.3% (165/225) at 1-month poststudy, but this increase in knowledge was not found to be statistically significant.

**Table 1:** Demographic characteristics of participants in the intervention group (n=225).

Demographic characteristic		n (%)
**Sex**		
	Male	70 (31.1)
	Female	155 (68.9)
**Religion**		
	Muslim	106 (47.1)
	Christian	97 (43.1)
	Druze	5 (2.2)
	Other	17 (7.6)
**Religiosity**		
	Very religious	23 (10.2)
	Religious	141 (62.7)
	Nonreligious	50 (22.2)
	No answer	11 (4.9)
**Year of study**		
	First	49 (21.8)
	Second	69 (30.7)
	Third	36 (16.0)
	Fourth, or higher	11 (4.9)
	Graduate level (master’s or doctoral level)	60 (26.7)
**Marital status**		
	Single	158 (70.2)
	Married	67 (29.8)

**Table 2:** Pre-/poststudy smoking knowledge and behavior of participants.

Question about smoking knowledge and behavior		Prestudy, n (%)	Poststudy, n (%)	χ^2^_1_	*P* value
**Did you smoke at least one cigarette during the last week? (n=225)**					
	Yes	50 (22.2)	45 (20.0)	0.1	.732
	No	175 (77.8)	180 (80.0)		
**In the past month, did you smoke nargila at least once a week? (n=225)**					
	Yes	131 (58.2)	50 (22.2)	60.6	.001
	No	94 (41.8)	175 (77.8)		
**Do you think it is safe to smoke cigarettes for only a year or two, as long as you quit after that? (n=225)**					
	Yes	156 (69.3)	165 (73.3)	0.9	.348
	No	69 (30.7)	60 (26.7)		
**Only smokers: In the past year, did you try to quit smoking? (prestudy n=50, poststudy n=45)**					
	Yes	29 (58)	33 (73)	2.5	.117
	No	21 (42)	12 (27)		
**Only smokers: Do you intend to quit smoking cigarettes in the next 6 months? (prestudy n=50, poststudy n=45)**					
	Yes	29 (58)	36 (80)	5.3	.021
	No	21 (42)	9 (20)		
**Only smokers: Have you received any support in trying to quit smoking? (prestudy n=50, poststudy n=45)**					
	Yes	34 (68)	34 (76)	0.7	.415
	No	16 (32)	11 (24)		

### Program Acceptability and Satisfaction

Participants expressed high satisfaction with the intervention (see Table 3). Of all the participants, 53.8% (121/225) reported that they were satisfied with the intervention, and 44.0% (99/225) were very satisfied with the program. Of all the participants, 94% (211/225) expressed that they would recommend the program to a friend.

**Table 3:** Acceptability and satisfaction of the Web-based tailored intervention (n=225).

Question about the tailored intervention		n (%)
**What is your satisfaction level regarding the Web-based tailored intervention?**		
	Very satisfied	99 (44.0)
	Satisfied	121 (53.8)
	Neutral	5 (2.2)
	Dissatisfied	0 (0)
	Very dissatisfied	0 (0)
**Would you recommend the Web-based smoking intervention to a friend?**		
	Yes	211 (93.8)
	No	14 (6.2)

### Focus Group Session Results

Five focus group sessions were held with a total of 56 individuals—35 (63%) females and 21 (38%) males—who completed the pre- and poststudy questionnaire. A focus group interview guide consisting of semistructured topics (shown in Textbox 1) was developed. However, since the goal of the focus group sessions was to give participants as much freedom as possible to express their opinions and views regarding the Web-based program, these semistructured questions were used only as a guide by the facilitator. The facilitator intervened with further exploratory questions only when the discussion reached a dead end. The facilitator diverged many times from the question pool to explore emergent themes and opinions that came up through the discussion.

**Focus group topics from the interview guide.**

Focus group topics:

1: Smoking information

2: Acceptability, feasibility, and availability of the program

3: Suggestions for improvement

4: Topics of interest

5: Educational health feedback received

6: Other health topics

7: Learning preferences

8: Satisfaction

The Internet was a predominant theme that arose when participants expressed how they usually received health information. To gain insight into whether participants felt the computer program was an acceptable, preferable, and useful tool, several questions were posed. The majority (50/56, 89%) of participants—males and females, as well as control and intervention group members—reported preference of the computer program over other traditional means of health education. However, more females (32/35, 91%) than males (17/21, 81%) indicated that they would likely use an online health intervention in the future. The following seven themes emerged when asked what they liked or preferred about the computer program: (1) interesting and easy to complete and understand, (2) educational videos, (3) easily accessible, (4) private and comfortable, (5) comparable to real life, (6) feedback, and (7) in Arabic. Similarly, the participants—intervention and control, alike—reported the feedback to be relevant, effective, clear and to the point, and interesting. The following statements demonstrate the predominant themes that resulted during the discussion regarding positive aspects of the Web program and feedback:

I usually get bored quickly when I participate in programs like this. Usually, the health education material is boring and irrelevant to my lifestyle. But this was different. The feedback, especially the videos, was very interesting and I actually learned something. I felt like the education given was specifically directed to me, telling ME exactly what I need to change and what I need to do to improve my health.

Participant, female

What I liked most was that I could access the program anytime and from anywhere. The first time, I completed it at a coffee shop. The second time, I did it while waiting for the bus at the bus station, and the last time I did it on campus. It was nice that in my spare time, and when I was ready and free, I could complete it.

Participant, male

I am a pretty shy person. I prefer to search the Internet. I am not always comfortable to talk about some things, such as what I eat or that I smoke, in front of my family doctor or nurse, so I never ask them questions. I always feel like they will look negatively upon me. With this, it was just me and the computer, and there was no one to judge me on how I choose to live my life.

Participant, female

All participants agreed that they would recommend the computer program to their friends and family, since it provides important information in a concise interesting way, is easily accessible, easy to understand, and does not require a lot of time. This strengthened the quantitative data regarding the program's acceptability. The majority (46/56, 82%) of participants agreed that even friends or family members who were not very familiar with using the computer could easily use and understand such a program. Most participants in the tailored-feedback intervention stated that they read at least 90% of the material presented.

The majority (49/56, 88%) of participants reported that the feedback regarding nargila smoking was most useful and interesting. During all sessions, participants agreed that there is a real lack of awareness and knowledge regarding nargila smoking. Furthermore, the majority (40/56, 71%) agreed that nargila smoking was socially and culturally acceptable. The following statements were made by participants:

Before reading the feedback on nargila smoking, I had no idea how dangerous it was. Everyone knows cigarettes are not healthy, but I didn’t realize that nargila was just as bad as (cigarettes) or even worse. No one has ever talked to me about health hazards associated with nargila smoking. To me, this is really scary since everyone today is smoking nargila. I know kids as young as 10 years who smoke nargila and their parents think it is ok. It’s socially acceptable, not like cigarettes. As a girl, I am not ashamed to smoke nargila in public but I would never smoke cigarettes in public or in front of my parents and family.

Participant, female

Nargila smoking just seems better for you than cigarette smoking. The tobacco is fruit flavored, the smell and taste of the smoke is very fruity. Also, it seems that with the water pipe, the water would clean out, or purify, the tobacco before you inhale it.

Participant, male

## Discussion

### Principal Findings

This study used mixed methodology to investigate the feasibility and acceptability of a Web-based intervention as a tool to increase knowledge and reduce cigarette and nargila smoking behavior among Arab college and university students in Israel. There were 225 participants who completed the prestudy questionnaire and the poststudy questionnaire after participating in the online tailored-feedback intervention. The findings from the study suggest that a tailored Web intervention was found interesting and acceptable among Arab university students and seems promising in reducing nargila smoking and increasing intention to quit smoking.

Based on previous findings in the literature, we originally hypothesized that a Web-based intervention would be an effective tool to decrease smoking behavior among the target population. However, this was only true for nargila smoking. The tailored intervention reduced nargila smoking from 58.2% at baseline to 22.2% at the 1-month follow-up.

The tailored intervention did not have a significant impact on reducing cigarette smoking. However, the cigarette smokers who participated in the tailored-feedback intervention reported a statistically significant increase in the intention to quit smoking (*P*=.021).

The program had a greater impact regarding changing nargila smoking. Unlike cigarette smoking, which is widely known by the general public to be dangerous to health, there is very little awareness among the target population regarding the heath impact of nargila smoking. From the focus group sessions, we learned more in-depth reasons as to why the Web-based intervention was so effective at reducing nargila smoking, but did not have any impact on cigarette smoking. It was evident that the harmful effects of cigarette smoking were recognized by all participants. However, this was not the case with nargila smoking, which is socially acceptable in Arab culture. The fruity smell and taste of the nargila smoke misled participants to believe it was not very harmful to health. Furthermore, it was clear that there was a lack of knowledge and awareness regarding nargila smoking. All participants reported that no one had previously talked to them about the harmful health hazards associated with nargila smoking, unlike cigarette smoking, which was thoroughly discussed in schools, through media campaigns, and advertisements.

Participants reported in the questionnaires, and emphasized during the focus group sessions, that they found the program to be acceptable, relevant, and interesting. Participants reported preference of the Web as an educational and data collection tool to more traditional strategies, such as printed materials. Almost all of the participants reported that they would recommend a similar program to a friend. Participants appreciated that the feedback they received was tailored to their responses. Similar findings throughout the literature have shown that participants preferred tailored feedback over general feedback because it is more relevant, interesting, and personal [22-30].

At the time the intervention was implemented (2007-2010), Web-based health programs were increasing in popularity around the world and nonexistent among our specific target population, Arab university students in Israel. Although the type of Web-based intervention presented is out of date in many technologically advanced countries, we believe it is still very important to present the findings of this basic technology. This is particularly true since there is a significant proportion of the general Arab population in Israel who lack access to more advanced technologies. Furthermore, such basic technologies remain unique and new to the specific target population at hand. However, with the increased use of mobile phone and other mobile technologies today, there is a need to develop and examine the effectiveness of more advanced technologies.

### Comparison With Prior Work

The results of our study are partially consistent with some studies in the literature. Our study found no significant changes in cigarette smoking among the target population at the 1-month poststudy session. This is consistent with findings from a randomized controlled trial (RCT) implemented by McKay et al that tested two Web-based smoking cessation interventions and failed to find any differences in smoking behavior at 3- and 6-month follow-up [31]. However, following participation in the tailored Web intervention, there were significant increases in the intention to quit smoking cigarettes, indicating that the tailored intervention may have had a positive impact. One systematic review concluded the evidence supporting the use of Web-based interventions for smoking cessation is insufficient in showing moderation of smoking behavior in adults, as well as insufficient in college students and adolescents [32].  On the other hand, another systematic review conducted by Civljak et al concluded that some Internet-based interventions can assist cigarette smoking cessation if programs last 6 months or longer, particularly those which are tailored to individuals [13,30]. The review found few trials that reported success rates for smoking cessation after 6 months or more, and other trials provided only limited evidence of long-term benefits of Web-based smoking cessation programs. However, it was found that the Internet-based programs may have an additional benefit when used alongside other interventions, such as nicotine replacement therapy or other pharmacotherapy [30]. A meta-analysis of 22 studies evaluating the effectiveness of Web- or computer-based smoking cessation programs indicated that there is sufficient clinical evidence to support the use of these programs for adult smokers [28].

In our study, the tailored-feedback Web intervention resulted in statistically significant reductions in nargila smoking (*P*=.001). To the best of our knowledge, this is the first Web-based program that has been developed to specifically change nargila smoking behavior, thus, we could not compare this study to others in the literature. However, if we consider nargila smoking to be smoking behavior in general, then our results are consistent with those found by other researchers indicating that Web-based programs are successful at effecting smoking cessation. [13,28,30].

### Study Limitations

There were several limitations in the study that must be considered. A major limitation was the small sample size of smokers, both at baseline and throughout the follow-up sessions, thus, a lack of statistical power. Despite recruiting an adequate sample size, there were a large number of participants that did not smoke from the outset. Since smoking was only one of four lifestyle behaviors examined in the program, it was not feasible for us to recruit only smokers. Of the 225 participants who completed the pre- and poststudy questionnaire, only 50 were cigarette smokers. With this small sample size, the statistical power of the study was low. In future studies, it would be more appropriate to recruit only cigarette and nargila smokers from the outset to guarantee that a larger sample of smokers remain at follow-up. Further research is needed to validate the effectiveness of the Web intervention with a larger sample of Arab-Israeli university students, using a rigorous research design, such as a randomized controlled trial. Since we do not have a control group, conclusions regarding the effect of the intervention cannot be made. Despite positive behavior changes regarding nargila smoking and intention to quit, we cannot directly state that these improvements were a result of the program.

Another limitation was that our findings were based on self-reports, which may have led to recall bias. However, because we used the same questions at all measurement sessions, this may not have influenced our data showing changes in behavior, and thus the effectiveness of the intervention. Despite sound theoretical underpinning and knowledge learned from previous work, there will always be limitations involved in using self-administered questionnaires. In future studies, self-reported changes should be compared with objective measures. It must be noted that outcomes of self-reported behavior and objective measures of the same type of behavior do not always match [33]. Consequently, additional research that utilizes measures of directly observed smoking behavior would strengthen the findings of this study and should be incorporated as part of future studies. Another aspect of the study that must be considered is the validation of the participants’ engagement and reading of the educational materials. During the focus group sessions, participants were also asked to report on how thoroughly they read the educational materials and if they found them to be interesting. Website analytics were used to assess when, and for how long, participants used the website. However, this does not provide enough accurate information regarding the validation of whether participants actually read the educational material.

The results obtained in this study are only applicable and generalizable to educated Arabs in Israel. It was assumed that if this program was not effective among the educated Arab population, then it would not be effective among the general population who may have less access to, and less experience using, the Internet. However, the distribution of education among participants is important. The participants included had various levels of education, including undergraduate students at college and university, as well as graduate students at the master's and doctoral levels. Since it was found that computer-based health interventions are a promising tool among educated Arabs, it could be an important strategy for change among the whole Arab population. Future research is necessary to test for applicability in other groups within the general Arab population, who may have lower education levels.

### Conclusions

Our findings revealed that a tailored Web-based program may be a promising tool to reduce nargila smoking among Arab university students in Israel. The tailored Web intervention was reported to be feasible, acceptable, relevant, and interesting. Future research is necessary to test for applicability in other groups within the general Arab population of Israel, who may have lower education levels. With the increased use of mobile phone and other mobile technologies today, there is also the need to develop and examine the effectiveness of more advanced technologies.

## Multimedia Appendix 1

CONSORT-EHEALTH checklist V1.6.2 [34].

## Abbreviations

QSIA: Questions Sharing and Interactive Assignments
RCT: randomized controlled trial
