# Supplementary material for: A Web-Based Program to Increase Knowledge and Reduce Cigarette and Nargila Smoking Among Arab University Students in Israel: Mixed-Methods Study to Test Acceptability
Source: J Med Internet Res. 2015 Feb 20;17(2):e39. doi: 10.2196/jmir.2988 (PMC4376148; doi:10.2196/jmir.2988)
Supplement: Supplementary file 1 [file jmir_v17i2e39_app1.pdf]

CONSORT-EHEALTH Checklist V1.6.2 Report

(based on CONSORT-EHEALTH V1.6), available at [http://tinyurl.com/consort-ehealth-v1-6].

2988

Date completed

9/28/2013 15:41:15

by

Jumanah Essa-Hadad

Effectiveness of a tailored vs. general feedback Web-based program as a health promotion tool to increase knowledge and improve smoking behaviors among Arab university students in Israel: A Randomized Controlled Trial

TITLE

1a-i) Identify the mode of delivery in the title

"web-based"

1a-ii) Non-web-based components or important co-interventions in title

Yes. "This educational material was also sent to the email address provided by the participant and was given at the baseline, as well as following each follow-up session."

1a-iii) Primary condition or target group in the title

"Arab university students in Israel"

ABSTRACT

1b-i) Key features/functionalities/components of the intervention and comparator in the METHODS section of the ABSTRACT

"A Web-based program, consisting of a questionnaire and feedback system on smoking (cigarette and Nargila) was developed. Arab university students were recruited to participate in a randomized controlled study, with pre-test–post-test control group design. The intervention group (n=326) received tailored feedback, while the control group (n=199) received general feedback. Follow-up was implemented at one and six months following baseline to determine if the intervention increased knowledge and affected behaviors."

1b-ii) Level of human involvement in the METHODS section of the ABSTRACT

"Arab university students were recruited to participate in a randomized controlled study, with pre-test–post-test control group design."

1b-iii) Open vs. closed, web-based (self-assessment) vs. face-to-face assessments in the METHODS section of the ABSTRACT

"A Web-based program, consisting of a questionnaire and feedback system on smoking (cigarette and Nargila) was developed. Arab university students were recruited to participate in a randomized controlled study, with pre-test–post-test control group design."

1b-iv) RESULTS section in abstract must contain use data

"A total of 263 participants, response rate of 50.1% (263/525), completed the intervention at baseline, 1 month, and 6 months follow-up post and were used for the comparative analysis."

1b-v) CONCLUSIONS/DISCUSSION in abstract for negative trials

"However, neither the tailored or general feedback interventions were successful at reducing cigarette smoking"

INTRODUCTION

2a-i) Problem and the type of system/solution

"Cigarette smoking is a serious public health problem in Israel, particularly among the male Arab population. Data from the Israeli Ministry of Health (2012) indicates that the highest smoking rates (age-adjusted) are among Arab men (52.8%) as compared to Jewish men (35.8%), whereas the rates were higher among Jewish women (25.1%) as compared to Arab women (10.7%) [2].

In addition to cigarette smoking, Nargila smoking is a phenomenon that has increased significantly over the years among the Arab population in Israel. A survey conducted by the Rikaz Database (2010) using a representative sample revealed that 60.5% of Arab Israeli's have tried using Nargila at least once and 9.3% use Nargila regularly, daily, and once a week [3]. When examining Nargila smoking according to age, it is evident that the largest percent (60.5%) belong to the 18-34 years age group, the age of most college/university students. Females hold a prominent place among Nargila smokers in comparison to their low rate among cigarette smokers, reaching about 19.6% of Arab Nargila smokers [3]."

2a-ii) Scientific background, rationale: What is known about the (type of) system

"Studies have evaluated the effectiveness, feasibility, and acceptability of using computer based health interventions to reduce smoking behavior {7,8,9,10,11]. A recent Cochrane review of 28 randomized and quasi-randomized trials on Internet-based interventions for smoking cessation found that some Internet-based interventions were effective at assisting smoking cessation [12]. The most promising interventions were ones that were interactive and tailored to the individuals. Computer-tailored health interventions can be defined as the adaptation of health education materials to one specific person through a largely computerized process [13]. Computer tailored health programs provide respondents with personalized feedback about their present health behavior and/or behavioral determinants, based on responses reported in a questionnaire. Computer tailored health interventions may offer the mix of high professional expertise typical of both mass communication and individual professional attention (tailored messages), while maintaining the cost-effectiveness of mass communication [14]. Compared with non-tailored messages, tailored health messages are more likely to be read and remembered, saved and discussed with others, perceived as interesting and personally relevant, and designed especially for them [15,16]."

METHODS

3a) CONSORT: Description of trial design (such as parallel, factorial) including allocation ratio

"The aim of this study was to test the hypothesis that a web-based health education program that provides tailored feedback can improve cigarette and Nargila smoking knowledge and behaviors of Arab college/university students in Israel."

3b) CONSORT: Important changes to methods after trial commencement (such as eligibility criteria), with reasons

Not applicable

3b-i) Bug fixes, Downtimes, Content Changes

Not applicable

4a) CONSORT: Eligibility criteria for participants

"The eligibility criteria included any Arab studying at a college or university in Israel, age 18 years and older, with access to the internet either at home or at their corresponding college or university, agreeing to provide informed consent to participation."

4a-i) Computer / Internet literacy

"The eligibility criteria included any Arab studying at a college or university in Israel, age 18 years and older, with access to the internet either at home or at their corresponding college or university, agreeing to provide informed consent to participation."

4a-ii) Open vs. closed, web-based vs. face-to-face assessments:

"All participants agreeing to the informed consent form were subsequently automatically allocated through use of an online random number generator into two groups: (1) tailored feedback intervention group (research group); and (2) general feedback intervention group (control group). They were asked to participate in the intervention at baseline, with follow-up after 1 month and 6 months. The intervention group received health education feedback that was tailored to their reported behaviors; the general feedback group (control) received general health educational materials (not tailored according to their responses) online following completion of the questionnaire."

4a-iii) Information giving during recruitment
"Potential participants received via email a letter in Arabic prior to the study, explaining its purpose and general procedure. All individuals who agreed to participate provided online consent prior to entering the online questionnaire. Once informed consent was given, participants were sent an email with an assigned user name and password, and a link to enter the online program."

4b) CONSORT: Settings and locations where the data were collected
"Male and female Arab students attending colleges and universities in Israel during 2007-2010 were recruited to participate in this study."

4b-i) Report if outcomes were (self-)assessed through online questionnaires
"The web program which was in the Arabic language consisted of two parts: (1) an interactive self-administered online questionnaire and (2) dissemination of health education material, either tailored or general."

"The primary outcome measure of effectiveness was self-reporting of cigarette and Nargila smoking behavior, and increases in knowledge at one month and six month follow-up."

4b-ii) Report how institutional affiliations are displayed
Not applicable, there were no sponsors to the program. The website that the participants accessed clearly showed that the study was affiliated with the University of Haifa. See screenshots.

5) CONSORT: Describe the interventions for each group with sufficient details to allow replication, including how and when they were actually administered

5-i) Mention names, credential, affiliations of the developers, sponsors, and owners
Not applicable, there were no sponsors to the program. The website that the participants accessed clearly showed that the study was affiliated with the University of Haifa. See screenshots.

5-ii) Describe the history/development process
"A second generation interactive computer tailored program was developed using the already existing QSIA system, an online assessment system that enables users, teachers, and students to generate, share, and manage knowledge items for learning, teaching, and assessment [18]. The validity and reliability of QSIA has been evaluated by previous research studies [19]."

"Before the web program was administered to the participants (pretest), it was pilot tested for appropriateness to (a) familiarize data collection personnel with the computer program (QSIA), (b) examine online interactions between the participants and researcher, (c) identify potential problems in the computer process, and (d) modify the questions appropriately to assure cultural appropriateness, user friendliness, and clarity. Twenty-five individuals, including teachers of the Arabic language, were sent the program and asked to provide feedback regarding clarity of the questions and health educational materials, grammar and spelling, and other administrative issues, such as if the questions are ordered correctly, and if the appropriate health education is received in the tailored intervention. After receiving the feedback from the pilot participants, either through email, phone conversation, or personal meeting, changes were made to the program before administering it to the participants. The data from the pilot study was not used in the analysis."

5-iii) Revisions and updating
Not applicable

5-iv) Quality assurance methods
"Before the web program was administered to the participants (pretest), it was pilot tested for appropriateness to (a) familiarize data collection personnel with the computer program (QSIA), (b) examine online interactions between the participants and researcher, (c) identify potential problems in the computer process, and (d) modify the questions appropriately to assure cultural appropriateness, user friendliness, and clarity. Twenty-five individuals, including teachers of the Arabic language, were sent the program and asked to provide feedback regarding clarity of the questions and health educational materials, grammar and spelling, and other administrative issues, such as if the questions are ordered correctly, and if the appropriate health education is received in the tailored intervention. After receiving the feedback from the pilot participants, either through email, phone conversation, or personal meeting, changes were made to the program before administering it to the participants. The data from the pilot study was not used in the analysis."

5-v) Ensure replicability by publishing the source code, and/or providing screenshots/screen-capture video, and/or providing flowcharts of the algorithms used
Supplementary files show screenshots.

5-vi) Digital preservation
Screenshots are provided.

5-vii) Access
"After individual randomized assignment of the participants to one of the groups, they were sent an introductory email in Arabic with basic information regarding the questionnaire, the link to the program and their assigned username and password to allow access to the program. Participants could access the program online from any computer with Internet access."

5-viii) Mode of delivery, features/functionalities/components of the intervention and comparator, and the theoretical framework
"The web program which was in the Arabic language consisted of two parts: (1) an interactive self-administered online questionnaire and (2) dissemination of health education material, either tailored or general. The interactive self-administered online questionnaire included a module on cigarette and Nargila smoking, that consisted of a series of 13 knowledge and smoking behavior questions, as well as module with 7 questions on demographic information about the participant.

All participants agreeing to the informed consent form were subsequently automatically allocated through use of an online random number generator into two groups: (1) tailored feedback intervention group (research group); and (2) general feedback intervention group (control group). They were asked to participate in the intervention at baseline, with follow-up after 1 month and 6 months. The intervention group received health education feedback that was tailored to their reported behaviors; the general feedback group (control) received general health educational materials (not tailored according to their responses) online following completion of the questionnaire.

After completion of the module, the web program was designed to immediately analyze responses and automatically displayed on the screen the health educational material for the participant to read, including a list of potential health risks based on the questionnaire responses and appropriate educational material, in Arabic, that aimed to increase health knowledge and promote positive health-related behaviors, specifically cessation of cigarette and Nargila smoking. The feedback that was given to the tailored group was done so according to the individual's perceived intention to change certain behaviors (according to the Transtheoretical Stages of Change Model) [20]. For example, if an individual in the tailored intervention group reported that they did not smoke, the feedback given was the following: Congratulations! You are not a smoker. You are protecting your health and have a less chance of developing certain diseases, like cancer and heart disease, in the future! Keep up the good work! On the other hand, if the participant reported that they smoke, they would receive the following recommendation: As a smoker, you are greatly increasing your risk of getting certain diseases, like cancers and heart disease, in the future? It is very important that you quit smoking now to protect your health. You can quit smoking by…(continue with recommendations). For those in the general feedback group, they were all given the same educational information on the harmful effects of cigarette and Nargila smoking, regardless of whether they smoked or not, and intention to quit.

This educational material was also sent to the email address provided by the participant and was given at the baseline, as well as following each follow-up session. As part of the feedback and recommendations, there were also links to educational YouTube videos that the participant's could watch."

5-ix) Describe use parameters
"They were asked to participate in the intervention at baseline, with follow-up after 1 month and 6 months."
5-x) Clarify the level of human involvement

5-xi) Report any prompts/reminders used
E-mail messages were used to remind individuals regarding follow-up dates for completing the questionnaire at 1 month and 6 months. Reminders were sent out by e-mail and/or via Facebook messages two weeks, one week, and again one day before students were expected to participate in each follow-up session.
5-xii) Describe any co-interventions (incl. training/support)
Not applicable
6a) CONSORT: Completely defined pre-specified primary and secondary outcome measures, including how and when they were assessed
The primary outcome measure of effectiveness was self-reporting of cigarette and Nargila smoking behavior, and increases in knowledge at one month and six month follow-up through completion of the self-administered questionnaire. Secondary outcome measures included intention to quit smoking, reason for wanting to quit, seeking professional help to quit smoking, and acceptability and preference of the program.
6a-i) Online questionnaires: describe if they were validated for online use and apply CHERRIES items to describe how the questionnaires were designed/deployed

6a-ii) Describe whether and how “use” (including intensity of use/dosage) was defined/measured/monitored

6a-iii) Describe whether, how, and when qualitative feedback from participants was obtained
"Following the online intervention, focus group methodology was utilized to examine participant’s personal perceptions and opinions regarding the acceptability, appeal, and effectiveness of such a web-based health program. Five focus group sessions were conducted based on procedures suggested by Krueger (1994) [17]. Three sessions were held with participants from the intervention group; two sessions were held with participants from the control receiving general online feedback. Due to sensitive issues that were expected to arise in the discussion, males and females were separated in the focus group session to allow for more comfort in discussion. Participants who completed all three tests as part of the web intervention were randomly recruited to participate in the sessions. To select participants, the researcher used an online random number generator and contacted those chosen via email or phone requesting their participation, until the needed number of participants were recruited. All five focus group sessions lasted approximately one and a half hours each. The sessions were moderated by an Arab professional group facilitator, familiar with the health field."
6b) CONSORT: Any changes to trial outcomes after the trial commenced, with reasons
Not applicable
7a) CONSORT: How sample size was determined
7a-i) Describe whether and how expected attrition was taken into account when calculating the sample size

7b) CONSORT: When applicable, explanation of any interim analyses and stopping guidelines
Not applicable
8a) CONSORT: Method used to generate the random allocation sequence
"All participants agreeing to the informed consent form were subsequently automatically allocated through use of an online random number generator into two groups: (1) tailored feedback intervention group (research group); and (2) general feedback intervention group (control group)."
8b) CONSORT: Type of randomisation; details of any restriction (such as blocking and block size)
"All participants agreeing to the informed consent form were subsequently automatically allocated through use of an online random number generator into two groups: (1) tailored feedback intervention group (research group); and (2) general feedback intervention group (control group)."
9) CONSORT: Mechanism used to implement the random allocation sequence (such as sequentially numbered containers), describing any steps taken to conceal the sequence until interventions were assigned
"All participants agreeing to the informed consent form were subsequently automatically allocated through use of an online random number generator into two groups: (1) tailored feedback intervention group (research group); and (2) general feedback intervention group (control group)."
10) CONSORT: Who generated the random allocation sequence, who enrolled participants, and who assigned participants to interventions
All participants agreeing to the informed consent form were subsequently automatically allocated through use of an online random number generator into two groups: (1) tailored feedback intervention group (research group); and (2) general feedback intervention group (control group).
11a) CONSORT: Blinding - If done, who was blinded after assignment to interventions (for example, participants, care providers, those assessing outcomes) and how
11a-i) Specify who was blinded, and who wasn’t
"Participants were not aware that the program provided different types of educational feedback (tailored or general)."
11a-ii) Discuss e.g., whether participants knew which intervention was the “intervention of interest” and which one was the “comparator”
" Participants were not aware that the program provided different types of educational feedback (tailored or general)."
11b) CONSORT: If relevant, description of the similarity of interventions
Not applicable
12a) CONSORT: Statistical methods used to compare groups for primary and secondary outcomes
"Descriptive statistics were first calculated to identify the characteristics of the participants and frequencies of behavior. Pearson's Chi-squared test was used to determine the significance of differences of socio-demographic characteristics between the intervention and control groups. To evaluate the effects on knowledge and behavior change as well as test for significant differences between the tailored and general feedback interventions, a repeated measure analysis of variance (ANOVA) was conducted, with time (baseline, 1 month follow-up, 6 month follow-up) as within-subjects factor and group (intervention and control) as between-subjects factor. Paired sample t-tests were used to determine if statistically significant changes occurred in behavior after participating in each intervention, comparing baseline to 1 month post. All data was analyzed using the statistical software, SPSS 19.0. Statistical significance was set at $p<0.05$ for all analyses.

Statistical Analysis of Qualitative Method
In order to analyze the focus group sessions, all sessions were audio recorded then transcribed. Following each focus group session, members of the research team conducted a debriefing to identify issues that may affect analysis, such as domineering or quiet members [21]. The completed transcriptions were compared with hand-written notes and any inaudible phrases or gaps in the tapes were made. Verification of the accuracy of the transcriptions was achieved by randomly cross-checking the transcripts against the tapes.
The results from the focus groups were analyzed using thematic analysis of the transcripts. This was done by organizing the statements from the focus group sessions into categories on the basis of themes (or concepts) for each of the 13 focus group questions that were asked. Concepts were then linked together as opposites or as sets of similar categories which are then made into theoretical statements. A selective coding template was developed based on major data themes; each them was given a different coding letter."
12a-i) Imputation techniques to deal with attrition / missing values

No

12b) CONSORT: Methods for additional analyses, such as subgroup analyses and adjusted analyses

No

RESULTS

13a) CONSORT: For each group, the numbers of participants who were randomly assigned, received intended treatment, and were analysed for the primary outcome

"There were 851 students that agreed to participate in the study. After analysis of eligibility, 113 participants were excluded after not meeting the eligibility criteria. Of the 738 that were randomized, 525 actually completed the baseline survey. The completion rate was 67% (n=352) at one month follow-up, and 50% (n=263) at 6 month follow-up for both arms."

13b) CONSORT: For each group, losses and exclusions after randomisation, together with reasons

"The completion rate was 67% (n=352) at one month follow-up, and 50% (n=263) at 6 month follow-up for both arms. There was no significant difference in dropout rates between the research and control groups. At 6 month follow-up the attrition rate was 52% (n=170) for the research group and 46% (n=92) in the control group."

13b-i) Attrition diagram

Participant flow chart clearly shows this.

"The completion rate was 67% (n=352) at one month follow-up, and 50% (n=263) at 6 month follow-up for both arms. There was no significant difference in dropout rates between the research and control groups. At 6 month follow-up the attrition rate was 52% (n=170) for the research group and 46% (n=92) in the control group."

14a) CONSORT: Dates defining the periods of recruitment and follow-up

"Male and female Arab students attending colleges and universities in Israel during 2007-2010 were recruited to participate in this study."

14a-i) Indicate if critical “secular events” fell into the study period

Not applicable

14b) CONSORT: Why the trial ended or was stopped (early)

Not applicable

15) CONSORT: A table showing baseline demographic and clinical characteristics for each group

Yes, Table 1: Demographic characteristics of participants

15-i) Report demographics associated with digital divide issues

No, not shown

16a) CONSORT: For each group, number of participants (denominator) included in each analysis and whether the analysis was by original assigned groups

16-i) Report multiple “denominators” and provide definitions

"We exposed 263 participants three times over a six month period to two different web interventions (tailored and general feedback) to assess changes in knowledge and behaviors related to smoking."

16-ii) Primary analysis should be intent-to-treat

17a) CONSORT: For each primary and secondary outcome, results for each group, and the estimated effect size and its precision (such as 95% confidence interval)

Tables 2 and 3 show changes from baseline to 6 month follow-up

17a-i) Presentation of process outcomes such as metrics of use and intensity of use

17b) CONSORT: For binary outcomes, presentation of both absolute and relative effect sizes is recommended

Not applicable

18) CONSORT: Results of any other analyses performed, including subgroup analyses and adjusted analyses, distinguishing pre-specified from exploratory

Not applicable. Sub-group analysis was done comparing males/females, religious/non-religious, and Muslim/Christians. However it is not presented in this paper, but will be presented in another article.

18-i) Subgroup analysis of comparing only users

19) CONSORT: All important harms or unintended effects in each group

No, not applicable

19-i) Include privacy breaches, technical problems

No, not applicable

19-ii) Include qualitative feedback from participants or observations from staff/researchers

"Twenty-five individuals, including teachers of the Arabic language, were sent the program and asked to provide feedback regarding clarity of the questions and health educational materials, grammar and spelling, and other administrative issues, such as if the questions are ordered correctly, and if the appropriate health education is received in the tailored intervention."

"Five focus group sessions were held with individuals who completed all three follow-up sessions. A total of 56 individuals (35 females, 21 males) participated in the five sessions. A total of 10 questions (Table 4) were asked during each focus group session to assess participant's acceptability and preference of the web-based program as a health promotion tool."

DISCUSSION

20) CONSORT: Trial limitations, addressing sources of potential bias, imprecision, multiplicity of analyses

20-i) Typical limitations in ehealth trials

"There were several limitations in the study that must be considered. The first is related to bias associated with self-reported and computerized methods. Despite sound theoretical underpinning and learning from previous work, there will always be limitations involved in using self-administered questionnaires. In future studies, there is a need to compare self-reported changes with objective measures. It must be noted that outcomes of self-reported behavior and objective measures of the same type of behavior do not always match [33]. Consequently, additional research that utilizes measures of directly observed behavior would strengthen the findings of this study and should be incorporated as part of future studies.
The use of technology and the Internet will always bring in another dimension and potential source of problems that can lead to non-completion, for example actual access to Internet and connection availability/problems. The use of technology may not be suitable for all, as there remains a proportion of the population who do not use, and do not intend to use computers (with or without the Internet). However, these individuals now appear to represent a diminishing proportion of the population while computer and Internet use continues to increase and become part of everyday life for most people.
Although Internet based methods are possibly not accepted by everyone yet, as each generation passes, and the younger generation who use computers/Internet more frequently become adults, these methods will become more and more accepted. Work with adolescents (the next generation of adults) has found that this group prefers assessment methods that incorporate new technology compared with more traditional methods [34]. Similarly, in this study which targeted younger adults (average age 25 years), the participants preferred the computer as a health promotion tool in comparison to other more traditional strategies. When using an Internet-based program, there is also the possibility of missing other groups, such as low socioeconomic status and low education, because of the current digital divides. However, these divides are continually narrowing.
Sampling bias is a problem in survey research as, no matter how many or who are invited to take part, respondents are ultimately those who 'choose' to complete the survey, resulting in a self-selected sample. Self-selection bias occurs as those who choose to respond to surveys tend to do so because they are affected by and/or interested in what the survey is asking about, or they are attracted to any incentives on offer [35]. In Web-based research, additional bias will be added if the target population is unrepresented by the Internet population.
The results obtained in this study are only applicable and generalizable to educated Arabs. Thus, the results found are therefore a lower-boundary estimate and thus even more useful. It was assumed that if this program is not effective among the educated Arab population, then it will not be effective among the general population. However, the distribution of education among participants is important. The participants included were from various levels of education, including undergraduate studies at college and university levels, as well as graduate master's and doctoral levels. Since it was found that computer based health interventions are a promising health promotion tool among educated Arabs, it could be an important health promotion strategy for change among the whole Arab population."

21) CONSORT: Generalisability (external validity, applicability) of the trial findings

21-i) Generalizability to other populations

"The results obtained in this study are only applicable and generalizable to educated Arabs. Thus, the results found are therefore a lower-boundary estimate and thus even more useful. It was assumed that if this program is not effective among the educated Arab population, then it will not be effective among the general population. However, the distribution of education among participants is important. The participants included were from various levels of education, including undergraduate studies at college and university levels, as well as graduate master's and doctoral levels. Since it was found that computer based health interventions are a promising health promotion tool among educated Arabs, it could be an important health promotion strategy for change among the whole Arab population. Future research is necessary to test for applicability in other groups within the general Arab population, who may have lower education levels."

21-ii) Discuss if there were elements in the RCT that would be different in a routine application setting

22) CONSORT: Interpretation consistent with results, balancing benefits and harms, and considering other relevant evidence

22-i) Restate study questions and summarize the answers suggested by the data, starting with primary outcomes and process outcomes (use)

"The findings from the study suggest that a tailored web-based intervention is acceptable and preferred as a health promotion tool among Arab university students and seems promising in reducing Nargila smoking, increasing intention to quit smoking cigarettes, and increasing professional help to quit smoking."

22-ii) Highlight unanswered new questions, suggest future research

"Future research is necessary to test for applicability in other groups within the general Arab population of Israel, who may have lower education levels."

Other information

23) CONSORT: Registration number and name of trial registry

Trial Registration ISRCTN59207794

24) CONSORT: Where the full trial protocol can be accessed, if available

Not applicable

25) CONSORT: Sources of funding and other support (such as supply of drugs), role of funders

This research did not have any funding.

X26-i) Comment on ethics committee approval

x26-ii) Outline informed consent procedures

X26-iii) Safety and security procedures

X27-i) State the relation of the study team towards the system being evaluated

No conflicts of interest.
